# Determinants and prevalence of zero-dose children in Somalia: Analysis of the 2020 Health Demographic Survey data

**DOI:** 10.1371/journal.pgph.0002612

**Published:** 2024-07-02

**Authors:** Said A. Mohamoud, Mohamed Abdullahi Ali-Salad, Ahmed Said Bile, Neha S. Singh, Amina J. Mahmud, Barni Nor

**Affiliations:** 1 Save the Children International, Mogadishu, Somalia; 2 Somali Institute for Development and Research Analysis (SIDRA), Garowe, Puntland, Somalia; 3 College of Arts, Humanities and Social Sciences, School of Social and Political Science, University of Edinburgh, Edinburgh, United Kingdom; 4 Global Health and Development Department, Faculty of Public Health and Policy, London School of Hygiene and Tropical Medicine, London, United Kingdom; 5 Uppsala University, Department of International Maternal and Child Health, Uppsala, Sweden; MRC Unit The Gambia at LSHTM: Medical Research Council Unit The Gambia at the London School of Hygiene and Tropical Medicine, GAMBIA

## Abstract

Despite global progress in childhood vaccination coverage, fragile and humanitarian countries, with high burden of infectious diseases, continue to report a significant number of zero-dose and under-vaccinated children. Efforts to equitably reach zero-dose children remain thus critical. This study assesses the prevalence and determinants of zero-dose children in fragile context of Somalia. We used secondary data from 2020 Somali Health and Demographic Survey (SHDS) to determine status of unvaccinated children aged between 12 to 23 months. Variables related to socio-demographic, household, health seeking, and community level factors were extracted from the SHDS data. Variables that were shown to be significantly associated with zero-dose children at p< 0.05 in the single logistic regression analysis were identified and included in a final multiple logistic regression analysis. A total of 2,304 women and their children aged between 12–23 months were used to determine the prevalence and determinants of zero dose children in Somalia. Approximately 60.2% of the children were zero dose children and did not receive any dose of the four basic routine vaccines. Children living in rural and nomadic areas were more likely to be zero dose (aOR 1.515, 95% CI: 1.189–1.93). Mother with primary education and above (aOR 0.519, 95% CI: 0.371–0.725), those who attended antenatal care (aOR 0.161, 95% CI: 0.124–0.209) and postnatal care (aOR 0.145, 95% CI: 0.085–0.245) and listen frequently to radio (aOR 2.212, 95% CI: 1.106–4.424) were less likely to have children with zero dose than with their counterparts. Majority of children under two years of age in Somalia are reported to be zero dose children. Context and population specific interventions that target vulnerable mothers and their children, in rural and nomadic areas, and from lower wealth quintile index families with no education and adequate access to antenatal and postnatal care remain critical.

## Introduction

The slow and poor progress of childhood vaccination in low-income countries has contributed to a significant pocket of zero dose children, or children who have not received any routine vaccination [1]. Recent pooled study, indicate that more than 17 million children globally are categorized as zero dose children [1]. These children are mainly concentrated in poor and disadvantaged households in urban slums, remote and rural areas, and conflict affected settings [2]. This finding is consistent with repeated cross-sectional study in India using data from 1992–2016 which found that zero dose children were predominantly concentrated in lowest wealth quintile households (15.3%), and mothers with no education (16.8%) [3]. Utazi et al., studied the characteristics and distribution of zero dose children in nine high priority low-and middle income countries (LMIC) over 10 year period, and found that the prevalence of zero dose children were associated with the use of maternal health services, maternal education and ethnicity [4].

Decades of conflict and recurrent humanitarian health emergencies has left Somalia on the top list of countries with high under five mortality and morbidity, most of which are attributed to vaccine preventable diseases [5]. The coverage of routine vaccination in Somalia remains significantly low, with less than 11 percent of children aged between 12–23 months fully vaccinated [6]. Unvaccinated (zero dose) children are assumed to live in poor urban slums, among Internally Displaced People (IDP), and in areas of outside of government’s control. These population groups are generally at risk of excess morbidity and mortality of vaccine preventable diseases due to their limited access to essential health and immunization services, displacement, severe food insecurity and malnutrition [7]. A recent cross-sectional study carried out in one district (Galkayo) in Somalia, affected by repeated community conflicts and which was the epicenter for polio outbreak in 2013, indicates that immunization coverage was associated with maternal education level (p = 0.0001), the place of delivery (p = 0.001) and the proximity to health facility (P = 0.003) [8]. A phenomenological study conducted by Abdullahi and colleagues [9], in the same district of Galkayo, documents several barriers to vaccine coverage including limited supplies and infrastructure, remote location, low awareness, low trust in vaccines which seem to have contributed to the low uptake of childhood vaccination in Galkayo district.

There is currently no study, to our knowledge, that assess the prevalence and determinants of zero children in Somalia. This study is part of a larger research project on zero dose children in crisis affected populations and which aims to assess governance and equitable delivery of vaccines to zero dose children. The findings of this study will contribute to the national immunization strategies and the newly launched Zero-dose Immunization Program (ZIP). It further informs the renewed global efforts in reaching zero dose children in fragile and humanitarian contexts and the goals of the Immunization Agenda 2030 (IA2030) and the ambitions of leaving no one behind [10–12].

## Methods

### Study setting

The primary data of the SHDS was collected during 2019–2020 from 18 pre-regions within Somalia. Each region was categorized into three strata of urban, rural, and nomadic, except for Banadir region which was considered fully urban. It is important to note however, that due to security concerns, the survey excluded all three strata of the Lower Shabelle and Middle Juba regions, as well as the rural and nomadic strata of the Bay region. As a result, the sampling frame for this investigation consisted of a total of 44 sampling strata. Although this survey did not consider displaced people as a specific category, they were included based on their urban or rural residing location.

### Data source

This study used secondary data from 2020 Somali Health and Demographic Survey (SHDS), which is conducted by the Somalia National Bureau of Statistics [6]. The 2020 SHDS is nationally representative survey of household samples. It is the first ever of such type of survey conducted in Somalia, and is planned to be conducted every 5 years. The SHDS offers comprehensive information about reproductive, maternal, child health and nutrition indicators, including immunization coverage. The methodology, sampling and questionnaire of the survey is extensively described in the SHDS 2020 report [13]. The variables of interest related to immunization were extracted from the child-focused dataset, KR data, which were extracted from data collected for the ever married women’s questionnaire of the SHDS, which collected data from Somali women of reproductive age (15–49 years).

### Study populations

The SHDS 2020 employed three stage stratified cluster sampling for urban and rural populations, and two stage three stage stratified cluster sampling for nomadic populations [13]. A representative sample of 16,360 households were selected, and 15,870 of those households were occupied. Of the occupied households, 15,826 households were successfully interviewed. From the interviewed households, 11,517 were ever-married women and of those ever-married women only 2,304 were with live child aged 12–23 months. We excluded women with dead children and those with children aged below 12 months and above 23 months, as those are not years of vaccination interest for children receiving any dose of four basic routine vaccines (BCG, Polio, DPT, Measles).

### Outcome variables

The outcome variable of analysis in this study was children with zero-dose vaccination status–those who had not received any dose of four basic routine vaccines (BCG, Polio, DPT, Measles). Zero-dose children were referred by IA2030 as those who did not receive even single dose of routine vaccines [10]. The SDHS survey collected childhood immunization information for children aged 12–23 months from their immunization cards and based on mother’s recall when the child did not receive a health card, or mothers were unable to show the immunization cards. In congruence with the recommendation of World Health Organization, we considered children with missing information on vaccination as not immunized [14].

### Explanatory variables

The independent variables of analysis in this study were selected based on the findings of previous studies [15, 16], authors’ existing knowledge of the Somali context, and availability of the variable of interest in the SDHS dataset. The selected maternal related variables were maternal age (<20, 20–34, 35–49), education level (no education, primary, secondary, and above), and marital status (married, divorce and widowed). The selected child factors were age (months), sex (male and female), and birth order (1, 2–3, 4–5, 6+).

The selected health seeking related variables were distance to health facility (big problem, no big problem), antenatal visits (none, 1, 2–3, 4+), institutional delivery (yes, no), postnatal care (yes, no), and the use of mass media–reading newspaper (yes, no), listening to radio (yes, no), and watching TV (yes, no). Residence (urban, rural, nomadic) and wealth index (lowest, second, middle, fourth, highest) were the chosen general variables.

### Statistical analysis

Due to the complex nature of SHDS sampling design, we weighted data using V005. Descriptive statistics of the background characteristics of study participants such as categorical data were summarized by using frequencies and percentages, while numerical variables were described by using means and standard deviation in the form of tables. In the single logistic regression analysis, variables significantly associated with zero dose status of children at the level of p<0.05 were identified, and then entered the multiple logistic regression analysis. In the final model, variable was significant if it had p-value of less than 0.05. All data management and statistical analysis were performed using SPSS version 24.

## Results

### Characteristics of the participants

A total of 2,304 women and their children aged 12–23 months were used to determine the prevalence and determinants of zero dose children in Somalia. More than two-thirds of women were aged 20–34 years (67.8%, n = 1563), 93.6% (n = 2156) were married and 82.3% (n = 1897) had no education. Thirty percent of mothers (n = 690) lived in urban areas and 13.1% (n = 301) belonged to the highest wealth index quintile.

Table 1 provides sociodemographic characteristics of women and their children. The majority of the women (63%, n = 1450) perceived distance to health facility as a problem, 73% (n = 1404) did not visit health facilities to receive antenatal care services, and health institutional delivery and the use of postnatal care services were only 15.2% (n = 346) and 12.4% (n = 286), respectively. The exposure of mothers to mass media were very limited–listening to radio was the most common media with only 5.3% (n = 122) of women reporting listen to it.

**Table 1:** Sociodemographic characteristics of mothers and their child aged 12–23 months, using the Somali Health and Demographic Survey 2020 (n = 2304).

Variable	Categories	X	%
**Maternal Variables**			
**Age**	<20	565	24.5
	20–34	1563	67.8
	35–49	176	7.6
**Marital status**	Married	2156	93.6
	Divorced	108	4.7
	Widowed	40	1.7
**Mother Education**	No Education	1897	82.3
	Primary	319	13.9
	Secondary	67	2.9
	Higher	20	0.9
**Child**			
**Sex**	Female	1229	53.3
	Male	1075	46.7
**Birth order**	1	452	19.6
	2 to 3	1661	72.1
	4 to 5	189	8.2
	6+	2	0.1
**Age (X ± SD, Min—Max)**	15.89 ± 3.519	12	23
**Health seeking behavior**			
**Distance to health facility**	Big problem	1450	63
	Not a big problem	851	37
**Antenatal care visits**	None	1404	73
	1	106	5.5
	2 to 3	412	21.5
	4+	1	0.02
**Institutional delivery**	Yes	346	15.2
	No	1932	84.8
**Postnatal care**	No	2017	87.6
	Yes	286	12.4
**Reading newspaper**	Yes	39	1.7
	No	2265	98.3
**Listening radio**	Yes	122	5.3
	No	2182	94.7
**Watching TV**	Yes	103	4.5
	No	2201	95.5
**Wealth**	Lowest	544	23.6
	Second	531	23.1
	Middle	451	19.6
	Fourth	476	20.7
	Highest	301	13.1
**Residence type**	Urban	690	30
	Rural	751	32.6
	Nomadic	863	37.5
**Vaccination status**	Not vaccinated	1388	60.2
	Vaccinated	916	39.8

The mean age of the children was 15.89 ± 3.519 months, 53.3% (n = 1075) were boys and majority were second and third born children (72.1%, n = 1661).

### Distribution and determinants of zero dose children

Zero dose children were found to belong to the most disadvantaged people and live in remote locations. Almost one third (31.1%, n =) of zero dose children were from the lowest wealth quantile as compared with 8.1% (n =) in the highest wealth quantile. Majority of women with zero dose children (90.6%, n =) were not educated, while 9.4% of mothers (n =) with zero dose children had primary and above education. Almost three quarters of zero dose children (73.8%, n =) lived in rural and nomadic settings, whereas 26.2% (n =) of zero-dose children were in urban locations.

In the single logistic regression, maternal education, perception of distance to health facility, antenatal and postnatal care use, exposure to media (reading newspaper, listening to radio, and watching TV), wealth index and residence were found to be significantly associated with zero dose children (p<0.05) (Table 2). When adjusted for potential confounders in the multiple logistic regression, maternal education level, the use of ANC and PNC services, listening to radio, wealth status and residence type remained significantly associated with zero dose children (p<0.05) (Table 2).

**Table 2:** Results of the single and multiple logistic regression to estimate the odds of children aged 12–23 months not being vaccinated (zero dose) in, using the Somali Health and Demographic Survey 2020 (n = 2304).

Variable	Category	Zero Dose		cOR	95% CI		P-value	aOR	95% CI		P-value
		X	%								
**Mother Education**	No Education	1257	90.60	1				1			
	Primary & above	130	9.40	0.24	0.191	0.302	0.000	0.519	0.371	0.725	0.000*
**Health facility distance**	Big problem	452	32.60	1				1			
	Not a big problem	935	67.40	0.623	0.525	0.741	0.0000	1.151	0.909	1.459	0.243
**ANC**	No	1054	89.20	1				1			
	Yes	127	10.80	0.108	0.086	0.137	0.000	0.161	0.124	0.209	0.000*
**PNC**	No	1305	94.10	1				1			
	Yes	82	5.90	0.22	0.168	0.289	0.000	0.145	0.085	0.245	0.000*
**Reading newspaper**	Yes	10	0.70	1				1			
	No	1377	99.30	4.168	2.04	8.516	0.000	1.01	0.314	3.249	0.987
**Listening radio**	Yes	45	3.20	1				1			
	No	1342	96.80	2.686	1.841	3.917	0.000	2.212	1.106	4.424	0.025*
**Watching TV**	Yes	10	2.50%	1				1			
	No	1377	97.50%	3.141	2.069	4.768	0.000	1.02	0.576	1.804	0.947
**Wealth**	Lowest	432	31.10%	1				1			
	Second	400	28.80%	0.785	0.59	1.046	0.098	0.825	0.586	1.161	0.269
	Middle	249	18.00%	0.32	0.242	0.422	0.000	0.615	0.431	0.878	0.007*
	Fourth	194	14.00%	0.178	0.135	0.235	0.000	0.317	0.222	0.451	0.000*
	Highest	112	8.10%	0.153	0.112	0.209	0.000	0.245	0.158	0.378	0.000*
**Residence type**	Urban	364	26.20%	1				1			
	Rural / Nomadic	1024	73.80%	1.556	1.299	1.864	0.000	1.515	1.189	1.93	0.001*

cOR Crude Odds Ratio, aOR Adjusted Odds Ratio, CI Confidence Interval

ANC Antenatal Care, PNC Postnatal Care

*p<0.05

Mothers with an education level of primary and above were less likely to have children with zero dose compared to unschooled mothers (aOR 0.519, 95% CI: 0.371–0.725; p-value = 0.000). Women who attended antenatal care services during pregnancy (aOR 0.161, 95% CI: 0.124–0.209; p-value = 0.000) and postnatal care services after delivery (aOR 0.145, 95% CI: 0.085–0.245; p-value = 0.000) were less likely to have zero dose children compared to women who did not attend these health services. Mothers who reported frequently listening to radios were found to have zero dose children relative to their counterpart mothers (aOR 0.452, 95% CI: 0.226–0.904; p-value < 0.025).

Children living in rural and nomadic locations were 1.5 times more likely to be zero dose relative to those children living in urban settings (aOR 1.515, 95% CI: 1.189–1.93; p-value = 0.001). The chance of having zero dose children decreased as the wealth quantile increased, with the lowest probability of a child being zero dose from a family with the highest wealth quantile index (aOR 0.25, 95% CI: 0.158–0.378; p-value < 0.000).

## Discussion

This study is to our knowledge, the first study in Somalia which examines the determinants and distribution and of zero dose children aged between 12–23 months. Maternal education is strongly associated with vaccination coverage. Mothers without education are more likely to have zero dose children compared to educated mothers, which is consistent with studies from other countries [17, 18]. Ghosh et al. used secondary data of 75,728 children from 34 Indian states and union territories, to examine the determinants for demand and supply of zero dose children in India. They found that mothers with no schooling were 2.3 times more likely to have zero dose children [19].

Access to essential healthcare services such as maternal and child health services remain a significant bottleneck for effective vaccination coverage. The findings of this are consistent with current capacity of the Somali health system, I which after decades of political conflict and instability remains weak and fragmented. Health and immunization provision is entirely donor dependent, and is characterized by limited and unpredictable financial resources, inadequate vaccine supply and demand, unclear governance structure and insufficient number of skilled human resources. Routine vaccination programs are generally implemented through routine immunization services, with limited or no specific attention and/or tailored supplementary programs for hard-to-reach and at risk population groups. On the contrary, polio programs are largely implemented through specific immunization campaigns.

Consistent with previous findings [20], mothers without any Antenatal care (ANC) visit are 80% more likely to have a zero dose child compared to a mother who had at least four ANC visits. Antenatal care visits marked health seeking behavior as mothers who visit health facility during pregnancy would later seek health care including vaccination of their child. A study in 33 Sub-Saharan African countries examined the determinants of zero-dose children over a decade, and found that mothers who attended health facilities during pregnancy and who received ANC were less likely to have zero-dose children [1]. Our study also found that mothers attending PNC services are more that 80% less likely to have zero dose children compared to mothers without any Postnatal care (PNC) visit. Antenatal care and PNC visits represent an important opportunity for mothers to receive information about childhood vaccination and to establish lasting relationship with health facilities. Healthcare workers can be trusted and primary sources of health information, including for child vaccination [21].

Mass media is a common strategy employed to improve the awareness of health services, including vaccinations [22, 23]. This study found that mothers who did not report listening to the radio are 60% more likely to have a zero dose child. Other sources of information, i.e., watching TV and reading newspaper, were found not to influence the zero dose children. A previous study reported the importance of radio-based health promotion interventions as it create community awareness, changes health seeking behavior and encourages mother to vaccinate their children [22]. Radio is the most commonly available and accessed mass media among nomadic and rural communities–communities with a higher prevalence of zero dose children in Somalia–so mothers in these areas rely on radio for health information. Low literacy and limited penetration of internet could impede the access of these potential source of information [6].

Consistent with previous studies, we found that zero-dose children in Somalia were strongly concentrated among the most disadvantaged communities, including nomadic and rural groups, and lowest quantile of wealth. There was vaccination disparity in household wealth, with the likelihood of zero dose children exponentially increasing from poorest to richest households. Rural and nomadic communities were 1.5 times more likely to have zero dose children compared to urban dwellers. Geographical location poses significant challenges for the delivery of immunization services in Somalia. It is estimated that 26% of Somali populations are nomadic [24]. These pastoral communities are mobile and on move, live in hard to reach areas, have less access to health services including immunization, and are majorly overlooked in humanitarian responses and developmental projects. Immunization in Somalia is predominantly implemented on a routine basis, with limited vaccines being delivered through supplementary immunization activities–which are much needed for zero-dose children and under-vaccinated communities. Mass immunization campaigns are largely undertaken for polio [7].

The strength of this study includes the use of the first ever 2020 SHDS which used nationally representative sample and can be generalizable across Somalia, to study zero-dose children.

## Study limitations

The study used data from the first ever demographic health survey in over 30 years in Somalia and has some limitations. Communal variables (internally displaced people, and insecurity) that could explain the determinants and distribution of zero dose children were not collected in the 2020 SDHS. Furthermore, 2020 SDHS data were collected prior to the COVID-19 pandemic, and as result, the findings of this study did not account for the decline in immunization rates due to COVID-19 related disruptions. Additionally, the potential sources of recall bias could introduce as a result of relying on mother’s recall of immunization history of her child if the vaccination card was unavailable. Prevalence of zero dose may also be associated with the availability and accessibility of health services to nomadic and rural populations, given the fragility of health system in Somalia. Security issues further exacerbate the situation as most of the communities reside in areas controlled by Al-Shabab, where the healthcare services are limited or nonexistent. Unfortunately, our study couldn’t capture the effect of supply on zero doses.

## Conclusion

Our study shows that mothers’ education, type of settlement, access to information and utilization of ANC and PNC are major factors contributing to the prevalence of zero-doze children in Somalia. Population specific interventions that target zero dose children remain critical and urgent, if Somalia is to reach the goals of the Immunization Agenda 2030. Somali health system is not designed to cater for nomadic and hard-to-reach population groups, despite the fact that these populations make up a significant proportion of the Somali population and their pattern of movement which is generally predictable (i.e. searching water and pasture for their animals). Further studies on how to effectively reach at risk populations, particularly mothers with no education, is essential if we are to reduce current literature gap and knowledge on zero dose children in Somalia.

## Supporting information

S1 Data Somali Health and Demographic Survey data set (SHDS). (SAV)
